# Effects of Elastic Therapeutic Taping on Knee Osteoarthritis: A Systematic Review and Meta-analysis

**DOI:** 10.14336/AD.2017.0309

**Published:** 2018-04-01

**Authors:** Xin Li, Xuan Zhou, Howe Liu, Nan Chen, Juping Liang, Xiaoyan Yang, Guoyun Zhao, Yanping Song, Qing Du

**Affiliations:** ^1^Xinhua Hospital Affiliated to Shanghai Jiao Tong University School of Medicine, Shanghai, China; ^2^Chongming Branch of Xinhua Hospital Affiliated to Shanghai Jiao Tong University School of Medicine, Shanghai, China; ^3^University of North Texas Health Science Center, Texas 76107, USA

**Keywords:** Kinesiotape, Knee osteoarthritis, Systematic review, Meta-analysis

## Abstract

Elastic therapeutic taping (ET) has been widely used for a series of musculoskeletal diseases in recent years. However, there remains clinical uncertainty over its efficiency for knee osteoarthritis (knee OA) management. To assess the effects of ET on patients with knee OA, we investigated outcomes including self-reported pain, knee flexibility, knee-related health status, adverse events, muscle strength, and proprioceptive sensibility. Ten databases including PubMed, EMBASE, Cochrane Library, CINAHL, Web of Science, PEDro, Research Gate, CNKI, CBM, and Wanfang were systematically searched. Eleven randomized controlled trials (RCTs) with 168 participants with knee OA provided data for the meta-analysis. Statistical significance was reported in four from five outcomes, such as self-related pain (during activity, MD -0.85, 95% CI, -1.55 to -0.14; *P* =0.02), knee flexibility (MD 7.59, 95% CI, 0.61 to 14.57; *P* =0.03), knee-related health status (WOMAC scale, MD -4.10, 95% CI, -7.75 to -0.45; *P* =0.03), and proprioceptive sensibility (MD -4.69, 95% CI, -7.75 to -1.63; *P* =0.003), while no significant enhancement was reported regarding knee muscle strength (MD 1.25, 95% CI, -0.03 to 2.53; *P* =0.06). Adverse events were not reported in any of the included trials. The overall quality of evidence was from moderate to very low. In conclusion, there is underpowered evidence to suggest that ET is effective in the treatment of knee OA. Large, well-designed RCTs with better designs are needed.

Osteoarthritis (OA), a chronic degenerative arthritis, is the most common form of all types of joint disease and mainly occurs in later life. The World Health Organization (WHO) forecasted that OA will become the fourth primary cause of disability by the year 2020 [1]. It has been reported that the burden of OA is physical, psychological, and socioeconomic; in particular, given the high frequency of OA worldwide, its economic burden is large [2]. While OA can and does affect many joints, knee OA is actually of particular interest to both investigators and clinicians as chronic knee pain is a common complaint in knee OA patients and it has been indicated as a major cause of limitations in mobility and daily activities [3]. Knee pain, decreased knee flexibility, and functional disability are a series of highly prevalent and common clinical characteristics during daily activities among patients with knee OA [4]. Previous studies have reported that pain and significant physical function decline have been associated with reduced muscle strength, poor proprioception, and impaired self-reported knee status [5,6]. Due to all these symptoms, it may be anticipated that knee instability will worsen over time. Conservative treatments (e.g., self-management programs, resistance strengthening, low-impact aerobic exercises, weight loss, whole-body vibration, and neuromuscular education) were recommended as clinical guidelines by the American College of Rheumatology (ACR) for knee OA. Clinically, the purpose of these treatments is to relieve pain, delay complications, and prevent disease progression [7,8].

Elastic therapeutic taping (ET), also known as kinesio taping (KT), which normally involves a combination of applying appropriate tension along the elastic therapeutic tape and placing the target muscle in a stretched position, is widely used as an interesting and relatively novel method for various clinical treatments. It was hypothesized to provide support and compression for muscular tension and joint distortions [9]. It was suggested that the therapeutic mechanism of ET should be related to the muscle fascia, tendon symptoms, and performance enhancement which were induced by improving circulation and lymphatic drainage^10^, but the application skills of ET have not been standardized and the elastic tension has been frequently applied depended on the clinicians’ experience so far. In addition, it has many proposed benefits, such as the provision of structural support, relief of swelling and inflammation, excitation and inhibition of muscle activity, and adjustment of blood and lymph flow [10,11]. In recent years, ET has gained popularity in clinical practice and sports settings for the prevention and treatment of musculoskeletal injuries, in particular following the 2008 Olympic Games [12], and it has been widely used in clinical practice by many physiotherapists worldwide.

Some reviews [13,14] have reported evidence that ET is superior to other forms of intervention for pain relief; however, a recent systematic review has highlighted that the current evidence cannot establish the superiority of ET in reducing disability when compared to either minimal or other forms of intervention in individuals with chronic musculoskeletal pain [15]. For individuals with knee OA, the self-reported pain was considered to be a limiting factor for physical function and strength, and as the disease progressed, a pain-weakness-pain vicious circle would be formed [16].

Among the different conservative non-pharmacological treatments used to treat knee OA, the application of ET has gained popularity [17]. Although there have been extensive efforts invested in evaluating the efficacy of ET on individuals with knee OA, a high-quality quantitative approach supporting its efficacy on pain, range of motion, functional disability, muscle strength, and proprioception is still lacking. Thus, this meta-analysis aimed to systematically review randomized controlled trials (RCTs) comparing the effect of ET intervention with other forms of intervention (including the comparisons of no treatment or sham/placebo KT) for the outcomes of self-reported pain, knee flexibility, knee-related health status, muscle strength, and proprioceptive sensibility in participants with knee OA.

## MATERIALS AND METHODS

### Literature Search

Our systematic review was conducted in accordance with the PRISMA (Preferred Reporting Items for Systematic Reviews and Meta-Analysis) guidelines [18]. For a completed PRISMA checklist, see Checklist S1. Systematic searches for literatures on the use of ET in knee OA populations were conducted of PubMed, EMBASE, Cochrane Library, CINAHL, Web of Science, PEDro, Research Gate, China National Knowledge Infrastructure (CNKI), Chinese BioMedical Literature Database (CBM), and Wanfang Database from inception to 28 September 2016. Both relevant conference and other literature that might have contained additional data were searched by search items such as “elastic taping”, “kinesiotape”, “knee osteoarthritis”, and “Randomized Controlled Trials”, and for details of the search strategies, see File S1. In addition, reference lists of included studies were manually screened, and we contacted authors for unpublished data related to our outcome measures.

### Inclusion Criteria

#### Types of studies

Only RCTs investigating ET versus other treatments (including all kinds of treatment expect ET such as general treatment, placebo KT or sham taping) for patients with knee OA were included. Neither publication dates nor language limits were required.

#### Types of participants

The participants in the trials included patients who met the American College of Rheumatology (ACR) criteria for diagnosing knee OA, which contains presence of pain in knee joint plus any three of six factors listed below:1) age more than 50-year-old; 2) presence of crepitus on active motion; 3) less than 30 min of morning stiffness; 4) bony tenderness; 5) bony overgrowth; 6) no palpable warmth of synovium. Patients exposed to analogous treatments (such as therapeutic KT or other adhesive taping) before the study were excluded unless an adequate washout period (not less than 1 week) was described.

#### Types of interventions

We included trials that compared ET with no treatment or other treatments (such as general treatment, placebo KT or sham taping) for knee OA. Studies in which the addition of ET over other interventions (intervention group) compared with other interventions only (control group) were also included.

#### Types of outcomes

As previously specified, the primary outcomes were measured including self-reported pain, knee flexibility, knee-related health status, and adverse events. Both symptomatic and asymptomatic adverse events were recorded. Secondary outcomes were muscle strength and proprioceptive sensibility. According to the intervention time, outcomes were divided into short-term (not longer than 6 days), mid-term (20 days), and long-term (21 days or more) follow-up.

**Figure 1.**
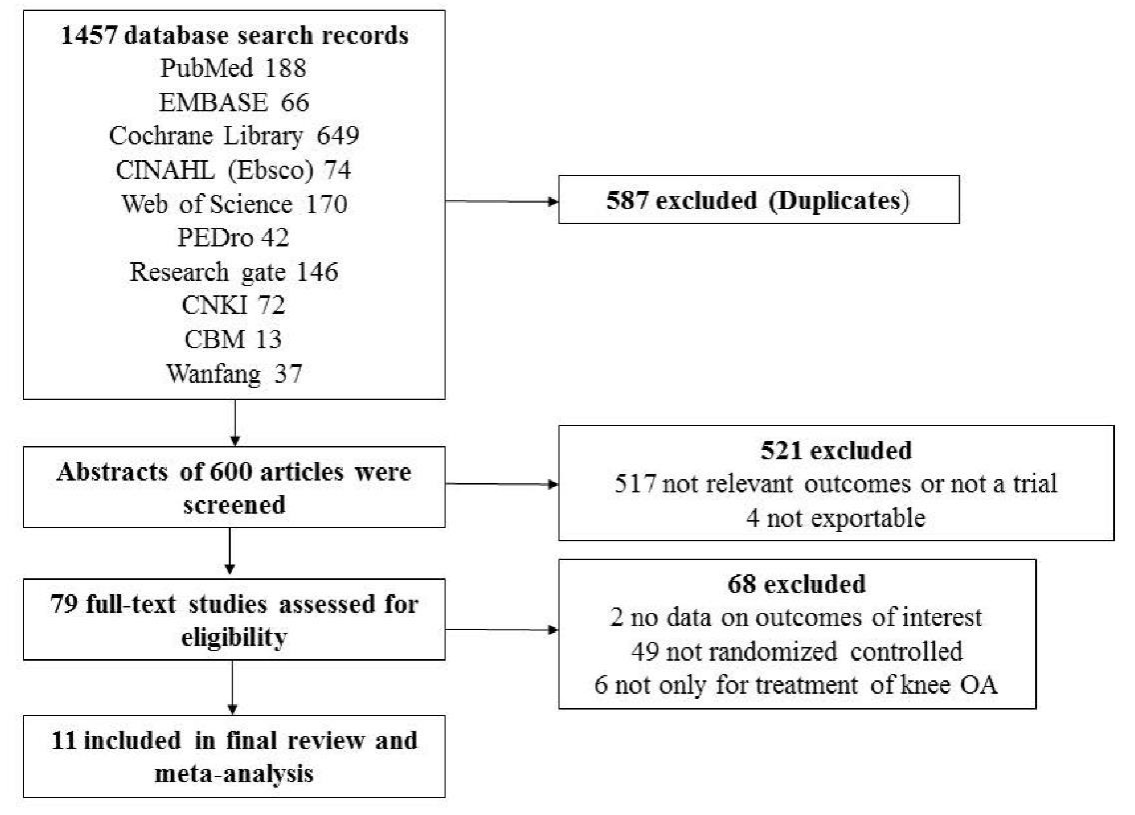
Review flow diagram

### Selection of Studies.

Two independent reviewers (Xin L and Qing D) independently sorted the articles for potentially relevant titles, and the judgment to include studies or not was made initially based on the study title and abstract. Abstracts of all identified records were each screened by two reviewers, and articles were retrieved in full whenever necessary. Disagreements were resolved through a consensus discussion with a third reviewer (Xuan Z) if necessary.

### Data Extraction

Information including the first author, publication year, characteristics of participants (such as age, gender, inclusion/exclusion criteria, sample size, and duration of complaint) and study design (such as intervention, control method, follow-up length, and outcome measures) were independently extracted by two reviewers (Xin L and Xuan Z), and then a cross-check was conducted for these data.

### Quality Assessment

Two independent reviewers (Xin L and Howe L) used Cochrane Collaboration’s tool for assessing risk of bias to identify the methodological quality of all included trials. Meanwhile, the methods described in each study were examined, and different bias domains including selection bias (random sequence generation, allocation concealment), performance bias (blinding of participants and personnel), detection bias (blinding of outcome assessments), attrition bias (incomplete outcome data), reporting bias (selective reporting), and other bias were assessed [19]. Risk of bias was evaluated in accordance with three grades: low risk, high risk, and unclear risk [20]. Any disagreements were resolved through discussion or third-party adjudication (Chen N).

**Figure 2.**
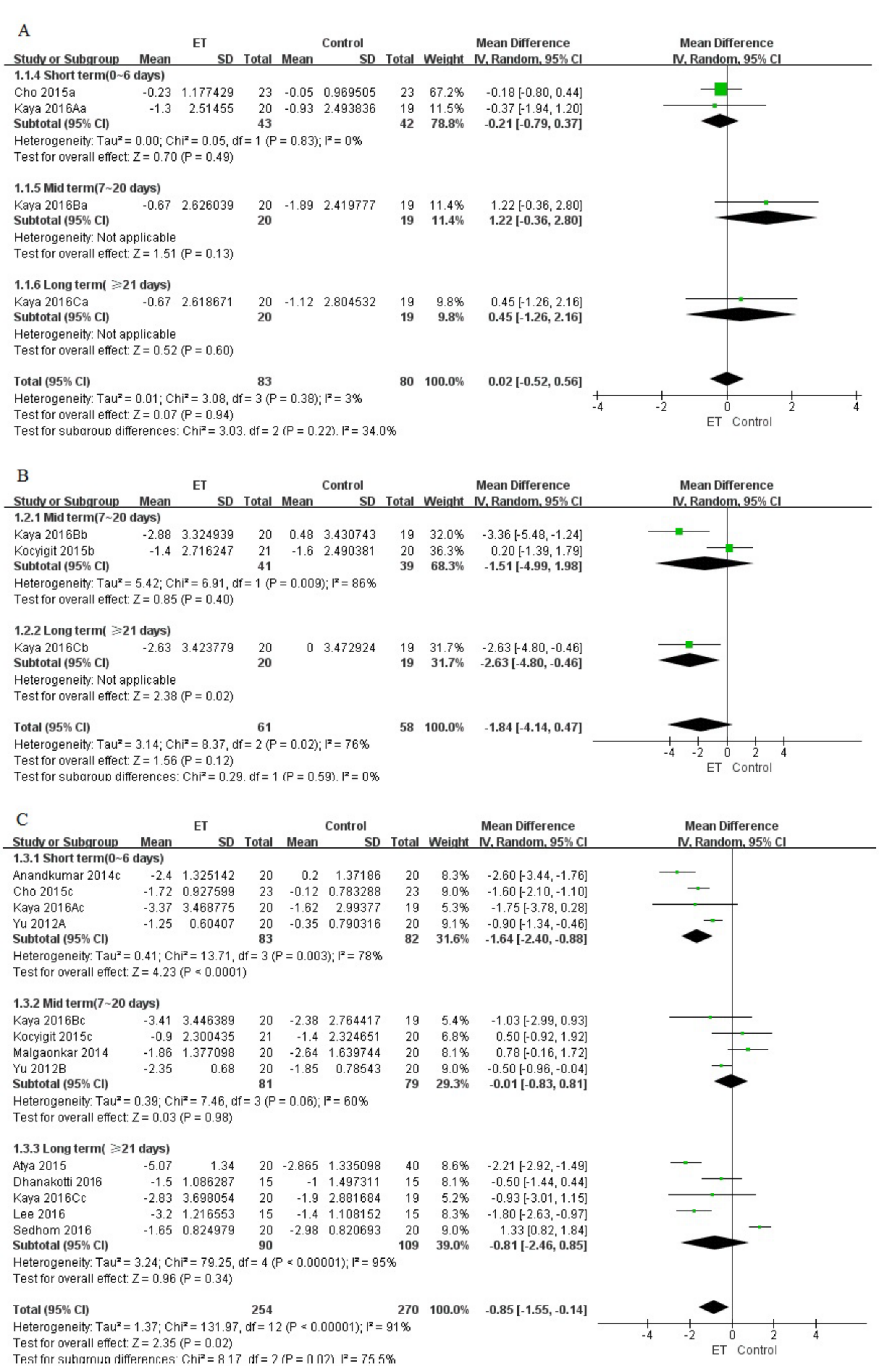
Self-reported pain (evaluated by VAS or NPRS) for ET compared with other forms of treatment. (A) pain at rest; (B) pain at night; (C) pain during activity.

### Statistical Analysis and Data Synthesis.

We contacted all corresponding authors to request the provision of unreported data which was required for our meta-analysis. The control conditions included other treatments, such as general treatment, placebo KT, and sham taping. The effects of ET on outcomes of interest were analyzed using Review Manager (RevMan 5.3). Synthesis of the data was performed using the random effects model if two or more trials evaluated the same outcome in the comparable groups. If two or more control groups performed various treatments in one trial, the data from the control groups were combined using the formula suggested by the *Cochrane Handbook for Systematic Reviews of Interventions* [19].

In addition, we estimated the mean difference (MD) with 95% CI in all outcomes. The *P* values were two-tailed, and significant differences are set at *P* <0.05. The proportion of variance in the pooled estimates caused by heterogeneity was evaluated by the following parameters: *I*^2^ index <25%, low heterogeneity, <75%, moderate heterogeneity, and ≥75%, high heterogeneity [21]. Funnel plot analysis was performed to visually assess the publication bias. Sub-analysis was conducted if the heterogeneities are significant.

The Grading of Recommendations Assessment, Development and Evaluation (GRADE) system provides grading criteria for assessing each of these factors including study design, study limitations (risk of bias), inconsistency, indirectness of study results, imprecision, and publication bias. It also specifies the quality of each primary outcome (such as self-reported pain, knee flexibility, knee-related health status, and adverse events) through categorizing studies into four levels (high, moderate, low, and very low) [22]. The judgment criteria are listed in Appendix S1.

**Table 1:** Characteristics of studies reporting the effectiveness of ET in knee OA and controls.

Article, Year	Patient Characteristics, Sample Size	Intervention	Duration of trial period	Outcomes/Time point/Effectiveness
[23] Yu (2012)	Source: 40 patients with knee OA (G1=20,G2=20) Mean age (SD): G1=51.12y (4.29), G2=50.97y(5.01)	G1: Therapeutic KT with less than 10% tension G2: CPT	G1: 24-hour each time, daily taping for 7 days G2: 20 min each time, daily treatment for 7 days	1. Pain intensity (VAS) / (Baseline, 3days^*^, 7days); 2. Functional disability (LI) / (Baseline, 3days^*^, 7days), (WOMAC) / (Baseline, 3days, 7days)
[24] Anandkumar (2014)	Source: 40 outpatients (G1=20, G2=20). Mean age (SD): G1=55.7y (5.8), G2=55.9y (5.0)	G1: Therapeutic KT with 50%-75% tension G2: Sham taping	Taping for 30 min	1. Peak isokinetic quadriceps torque (concentric and eccentric at angular velocities of 90° per second and 120° per second) / (Baseline, 30 min^*^); 2. Physical function (SSCT) / (Baseline, 30 min^*^); 3. Pain intensity (VAS) / (Baseline, 30 min^*^)
[25] Cho (2015)	Source: 46 volunteer subjects with knee OA (G1=23, G2=23). Mean age(SD):G1=58.2y(4.5), G2=57.5y (4.4)	G1: Therapeutic KT with 15%-25% tension G2: Sham taping	Taping for 60 min	1. Pain-free ROM of the knee joint (Active ROM) / (Baseline, 60 min^*^) 2. Pain intensity at rest (VAS), (PPT in quadriceps), (PPT in tibialis anterior) / (Baseline, 60 min); 3. Pain intensity during walking (VAS), (PPT in quadriceps), (PPT in tibialis anterior) / (Baseline, 60 min^*^); 4. Proprioceptive acuity (AJPR at 15, 30, and 45 degrees) / (Baseline, 60 min^*^)
[26] Kocyigit (2015)	Source: 41 outpatients with knee OA (G1=21, G2=20). Mean age(SD):G1=52y(7.5), G2=52y (10)	G1: Therapeutic KT with 25% tension G2: Sham taping	Taping was repeated every 4 days, 3 times in total	1. Functional disability (LI) / (Baseline, 12 days); 2. Quality of life (NHP pain score, NHP physical activity score, NHP sleep score, NHP social isolation score, NHP total score) / (Baseline, 12 days); 3. Quality of life (NHP energy score) / (Baseline, 12 days^*^);4. Pain intensity with activity and at night (VAS) / (Baseline, 12 days);
[27] Wageck (2016)	Source: 76 outpatients with knee OA (G1=38, G2=38). Mean age(SD):G1=69.6y(6.9), G2=68.6y (6.3)	G1: A multi-layer KT application G2: Sham taping	Taping for 4 days, follow -up for extra 15 days	1. Muscle strength (Knee extensor and flexor isokinetic concentric strength)/ (Baseline, 4 days, 19 days); 2. Pain intensity (PPT) / (Baseline, 4 days, 19 days); 3. Functional disability (WOMAC) / (Baseline, 4 days, 19 days); 4. Lower limb volume/ (Baseline, 4 days, 19 days); 5. Perimeter of the limb/ (Baseline, 4 days, 19 days); 6. Physical function (LKSS) / (Baseline, 4 days, 19 days)
[28] Lee (2016)	Source: 30 elderly patients with knee OA (G1=15, G2=15). Mean age(SD):G1=72.0y(4.0), G2=73.1y (5.8)	G1: KT G2: CPT	3 times/week for 4 weeks.	1. Pain intensity (VAS) / (Baseline, 4 weeks^*^); 2. Functional disability (K-WOMAC) / (Baseline, 4 weeks^*^); 3. Pain-free ROM of the knee joint (Passive ROM) / (Baseline, 4 weeks^*^);
[29] Dhanakotti (2016)	Source: 30 patients with knee OA(G1=15, G2=15). Mean age(SD):G1=51.73y(5.10), G2=51.26y (4.86)	G1: KT with 40% stretch of its maximal length+ CPT G2: CPT	3 times/week for 3 weeks	1. Pain intensity (NPRS) / (Baseline, 3 weeks^*^); 2. Muscle strength (Maximum isometric force of quadriceps) / (Baseline, 3 weeks^*^); 3. Functional disability (m.WOMAC) / (Baseline, 3 weeks^*^);
[30] Kaya (2016)	Source: 39 outpatients with knee OA (G1=20, G2=19). Mean age(SD): G1=52y (7.5), G2=52y (10)	G1: Therapeutic KT with 25% tension G2: Placebo KT	12 to 16 days in total	1. Pain intensity (VAS during activity), Functional disability (ALF-walking) / (Baseline, after the initial KT, after the third KT^*^, 1 month follow-up^*^), 2. Pain intensity (VAS at night), Pain-free ROM of the knee flexion (Active ROM) / (Baseline, after the initial KT, after the third KT, 1 month follow-up^*^), 3. Pain intensity (VAS at rest), Functional disability (WOMAC), Pain-free ROM of the knee and hip joints (Active ROM except knee flexion), Muscle strength (Maximum isometric force of iliopsoas, gluteus medius, quadriceps, and hamstring muscles)/ (Baseline, after the initial KT, after the third KT, 1 month follow-up)
[31] Atya (2015)	Source: 60 outpatients with knee OA (G1=20, G2=20, G3=20) Mean age (SD): G1=38.7y (7.7), G2=38.6y (7.5), G3=37.6y(5.6)	G1: TEP G2: TEP+KT G3: TEP+ST	TEP/ST: 3 times /week for 8 weeks KT: wear tape for 2 days and return for review after 24 hours removing tape	1. Pain intensity (VNS) / (Baseline, 8 weeks^*^); 2. Proprioceptive acuity (AJPR) / (Baseline, 8 weeks); 3. Physical function (AIFAS) / (Baseline, 8 weeks)
[32] Sedhom (2016)	Source: 40 females with knee OA from outpatient (G1=20, G2=20) Mean age(SD): G1=48.7y (5.82), G2=49.25y (5.82)	G1: HP+SEP+ KT G2: HP+SEP+Aescin and Diethylamine Salicylate gel PH with PUT	3 times /week for 4 weeks	1. Pain intensity (VAS) / (Baseline, 4 weeks) 2. Proprioceptive accuracy (AJPR) / (Baseline, 4 weeks) 3. Pain-free ROM of the knee joint (Active ROM) / (Baseline, 8 weeks)
[33] Malgaonkar (2014)	Source: 40 subjects with knee OA (G1=20, G=20). Mean age(SD): G1=53.5y (2.21), G2=52.95y (2.25)	G1: Therapeutic KT with 25% tension G2: MWM	3 times/week for 2 weeks	1. Pain intensity (VAS) / (Baseline, 2 weeks) 2. Functional disability (WOMAC) / (Baseline, 2 weeks)

OA, Osteoarthritis; KT, Kinesio taping; CPT, Conventional physical therapy; VAS, Visual analog scale; WOMAC, The Western Ontario and McMaster Universities Osteoarthritis Index; KWOMAC, Korean Western Ontario and McMaster Universities Osteoarthritis Index; m.WOMAC, The modified western Ontario and McMaster Universities Osteoarthritis Index; LI, Lequesne index; SSCT, Standardized Stair Climbing Task ;ROM, Range of motion; NHP, Nottingham Health Profile; PPT: Pressure pain threshold; LKSS, Lysholm Knee Scoring Scale; NPRS, Numeric pain rating scale; VNS, Visual numerical scale; ALF, the Aggregated Locomotor Function; TEP, Traditional exercise program;ST, Sensory motor training;AJPR, Active joint position reproduction; AIFAS, Arthritis impact functional assessment scale; HP, Hot packs; SEP, selected exercise program; PUT, Pulsed ultrasound therapy; MWM, Mulligan’s Movement with Mobilization;

* Means a significant difference compared with the control group(s).

## RESULTS

### Study Characteristics.

Our initial search yielded 1457records. After removing 587 duplicates, abstracts of 600 articles were screened by two independent reviewers. Of these articles, 79 were selected for detailed inspection. Subsequently, 68 articles were excluded for the following reasons: no data on outcomes of interest, not RCTs, and not only for treatment of knee OA. The remaining 11 RCTs covering 482 patients with valid outcome data met the inclusion criteria and were included in this analysis. The details of identifying these studies from initial publication searches to final inclusion are illustrated in Fig. 1.

Eleven RCTs with the purpose of examining the efficacy of ET on knee OA [23-33] were included in our meta-analysis, and a summary of their characteristics is shown in Table 1. The trials were published in English or Chinese from 2010 to 2016. All of them have made use of the KT and the time of intervention ranged from 30 minutes to 56 days (median: 15 days). Participants in five trials were graded II-III evaluated by Kellgren-Lawrence Scale, while other six reported no details on the severity of knee OA. Among the trials, three trials were from India [24, 29, 33], two trials were from Turkey [26, 30], two trials were from Egypt [31, 32], and the four other studies were from China [23], South Korea [25], Republic of Korea [28] and Brazil [27]. In the experimental groups, five studies used ET with no more than 25% tension (range from 0% to 25%, median: 15%) [23, 25, 26, 30, 33]; two studies used ET with tension ranging from 40% to 75% [24, 29]; and the other four studies [27, 28, 31, 32] did not mention the details of tension. Regarding controls, four studies [24-27] used sham taping, one used placebo ET [30], and the other studies used treatments such as conventional physical therapy, sensory motor training and so on [23, 28, 29, 31-33].

**Figure 3.**
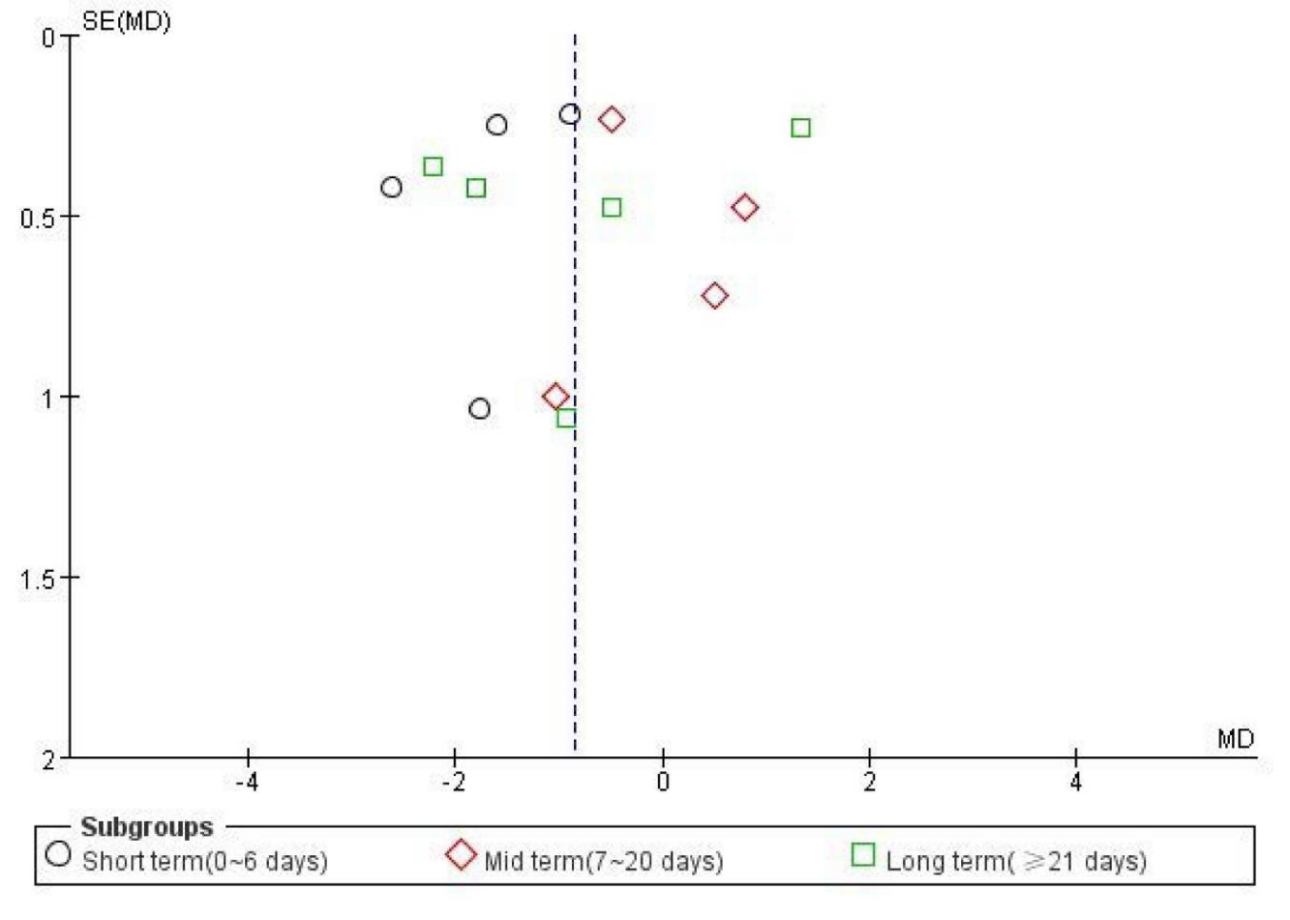
The funnel plot regarding self-reported pain during activity.

### Methodological Quality of Included Trials.

Following recommendations, the Cochrane Collaboration’s Tool of assessing risk of bias was used in each included trial for methodological assessment. One (9.09%) trial [28] reported no information about the random sequence generation; three (27.27%) trials [24, 29, 33] provided no information about allocation concealment, which resulted in unclear risk of selection bias. The blinding of participants and personnel was used in six studies [24-27, 30, 31], while 4 of the 11 included trials did not meet the requirements for the blinding of assessors [23, 28, 32, 33]. Incomplete outcome data were reported as high or unclear risk of bias in four trials [25, 27, 30, 31]. Furthermore, all the included trials were free of selective outcome reporting (Table 2).

### Effectiveness

Included studies which reported the effects of ET for knee OA were estimated by using five outcome measures, as none of the included trials reported adverse events. Other primary and secondary outcomes were listed as follows:

**Table 2:** The Cochrane Collaboration’s tool of assessing risk of bias for methodological assessment.

Author, Year	Random sequence generation	Allocation concealment	Blinding of Participants and personnel	Blinding of outcome assessments	Incomplete outcome data	Selective reporting	Other bias
[23] Yu, 2012	Low	Unclear	Unclear	Unclear	Low	Low	Low
[24] Anandkumar, 2014	Low	Low	Low	Low	Low	Low	Low
[25] Cho, 2015	Low	Low	Low	Low	High	Low	High
[26] Kocyigit, 2015	Low	Low	Low	Low	Low	Low	Low
[27] Wageck, 2016	Low	Low	Low	Low	High	Low	High
[28] Lee, 2016	High	Unclear	Unclear	Unclear	Low	Low	High
[29] Dhanakotti, 2016	Low	Low	High	Low	Low	Low	Low
[30] Kaya, 2016	Low	Low	Low	Low	High	Low	High
[31] Atya, 2015	Low	Low	Low	Low	Unclear	Low	Unclear
[32] Sedhom, 2016	Low	Unclear	Unclear	Unclear	Low	Low	High
[33] Malgaonkar, 2014	Low	Low	Unclear	Unclear	Low	Low	Unclear

#### 1. Primary Outcomes

##### (1) Self-reported Pain

The visual analog scale (VAS) and numeric pain rating scale (NPRS) were used to assess self-reported pain before and after intervention at rest [25, 30], at night [26, 30], or during activities [23-26, 28-33] such as sitting, walking, squatting, or during a standardized stair climbing test. It is certain that high correlations exist between these two different measures (*r*=0.957, *P*<0.0001) [34].

**Figure 4.**
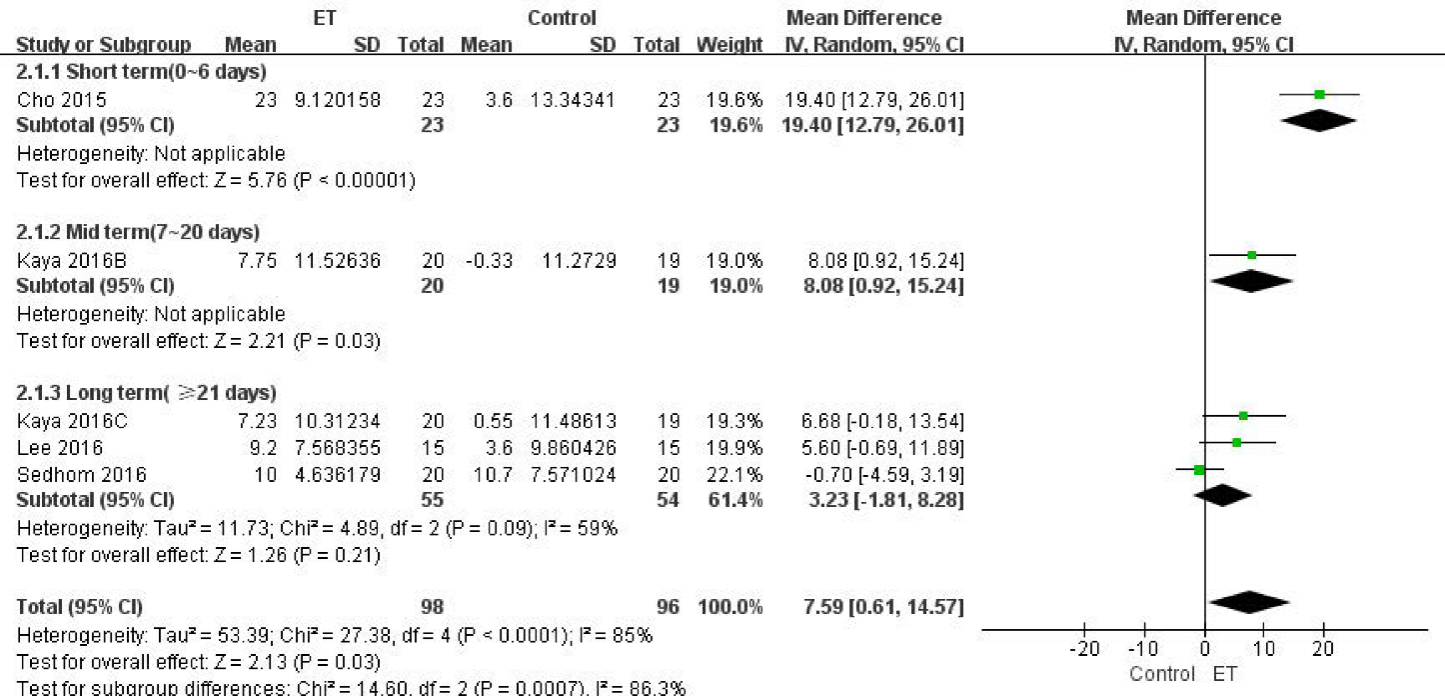
Knee flexibility (evaluated by knee ROM) for ET compared with other forms of treatment.

Among these trials, 2 (85 patients) reported pain at rest [25, 30], 2 (80 patients) reported pain at night [26, 30], and 10 (406 patients) reported pain during activity [23-26, 28-33]. We found significant improvements with ET for self-reported pain relief during activity (MD -0.85, 95% CI, -1.55 to -0.14; *P*=0.02; *P* for heterogeneity<0.00001, *I^2^*=91%), but the difference between groups was clinically not much significant with Minimal Clinical Important Difference (MCID) of 1.5-2.0 mm required to detect treatment effects on pain in individuals with knee OA (converting all to 0-10 scale) [39]. Moreover, no statistical or clinically significant difference in pain relief was found at rest (MD 0.02, 95% CI, -0.52 to 0.56; *P*=0.94; *P* for heterogeneity=0.38, *I^2^*=3%) or at night (MD -1.84, 95% CI, -4.14 to 0.47; *P*=0.12; *P* for heterogeneity=0.02, *I^2^*=76%) (Fig. 2). In addition, the funnel plot had an asymmetrical distribution regarding self-reported pain during activity, which suggested there would be a high risk of publication bias according to the shape of the funnel plot (Fig. 3).

##### (2) Knee Flexibility

The knee flexibility in patients with knee OA was assessed by measuring the knee range of motion (ROM) using a goniometer in four studies [25, 28, 30, 32]. All the measurements were pain free, and the average value of three measurements was obtained. Cho et al [25] and Kaya et al [30] showed the short-term and mid-term effects, respectively. Three studies reported data at long-term follow-up period [28, 30, 32]. Although we failed to find any difference between two groups in long-term comparison, a significant increase in knee ROM was found in the ET group (MD 7.59, 95% CI, 0.61 to 14.57; *P*=0.03; *P* for heterogeneity<0.0001, *I^2^*=85%) with overall data consisting of three subgroups (Fig. 4).

**Figure 5.**
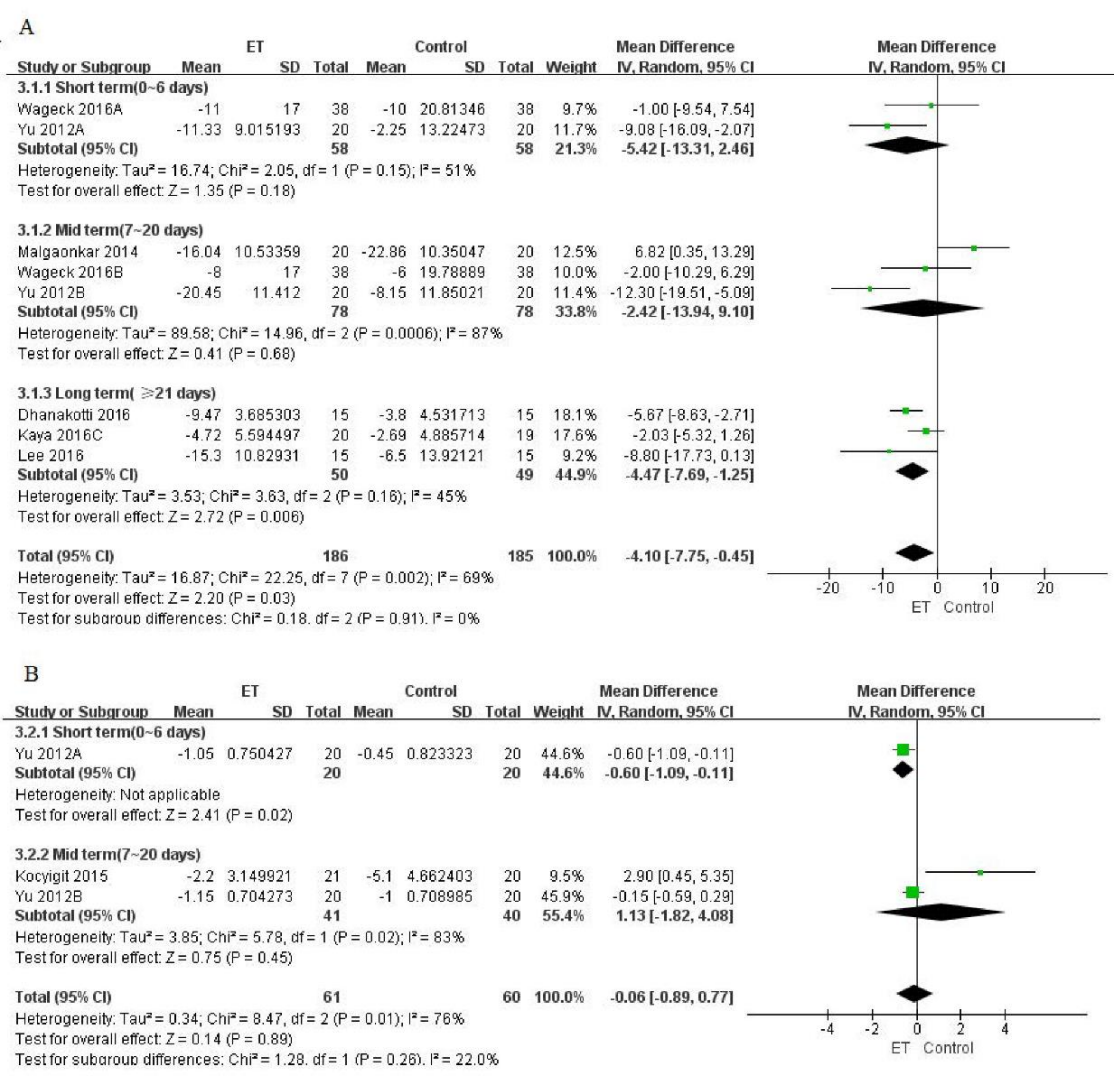
Knee-related health status (evaluated by WOMAC or LI scales) for ET compared with other forms of treatment. (A) WOMAC; (B) LI.

##### (3) Knee-related Health Status

Knee-related health status was assessed by either Western Ontario or McMaster Universities Osteoarthritis Index (WOMAC) or Lequesne Index (LI) scales. Among trials which reported WOMAC scales, two (116 patients) [23, 27] reported data at the short-term follow-up period, another three trials (166 patients) [23, 27, 33] at the mid-term, and three (99 patients) [28-30] at the long-term follow-up period. We found significant improvements with ET for WOMAC assessment in both long-term (MD -4.47, 95% CI, -7.69 to -1.25; *P*=0.006; *P* for heterogeneity=0.16, *I^2^*=45%) and total (MD -4.10, 95% CI, -7.75 to -0.45; *P* =0.003; *P* for heterogeneity=0.002, *I^2^*=69%) effects (Fig. 5A), and the difference between groups was clinically significant with MCID for WOMAC global and subscale scores ranged from 0.67-0.75 for improvement in individuals with knee OA[40]. Although Yu et al have reported a significant improvement of LI scales in the short-term period (MD -0.6, 95% CI, -1.09 to -0.11; *P* =0.02) [23], no significant difference was found either in mid-term (MD 1.13, 95% CI, -1.82 to 4.08; *P*=0.45; *P* for heterogeneity=0.02, *I^2^*=83%) or overall effects(MD -0.06, 95% CI, -0.89 to 0.77; *P*=0.89; *P* for heterogeneity=0.01, *I^2^*=76%) (Fig. 5B).

#### 2. Secondary Outcomes

##### (1) Knee muscle strength

**Figure 6.**
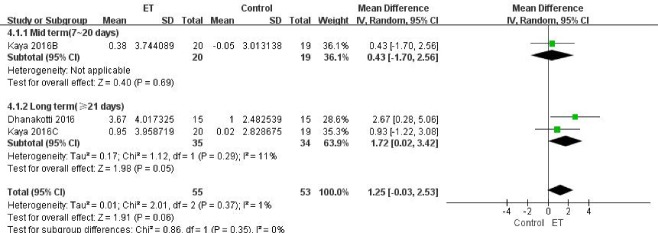
Knee muscle strength (evaluated by maximum isometric force of quadriceps) for ET compared with other forms of treatment.

Only 2 of the 11 included studies reported the maximum isometric force of quadriceps measured by a handheld dynamometer (HHD) [29, 30]. During the measurement, patients were placed in a high sitting position, and the HHD was placed distally to the knee joint. Therapists then asked the patients to isometrically extend the knee against the HHD, and the isometric force was recorded. The same test was repeated for three trials and the average score was recorded. Overall, no significant difference between ET and other treatments was observed for muscle strength from trials consisting of 108 patients (MD 1.25, 95% CI, -0.03 to 2.53; *P* =0.06; *P* for heterogeneity=0.37, *I^2^*=1%) (Fig. 6).

##### (2) Proprioceptive Sensibility

**Figure 7.**
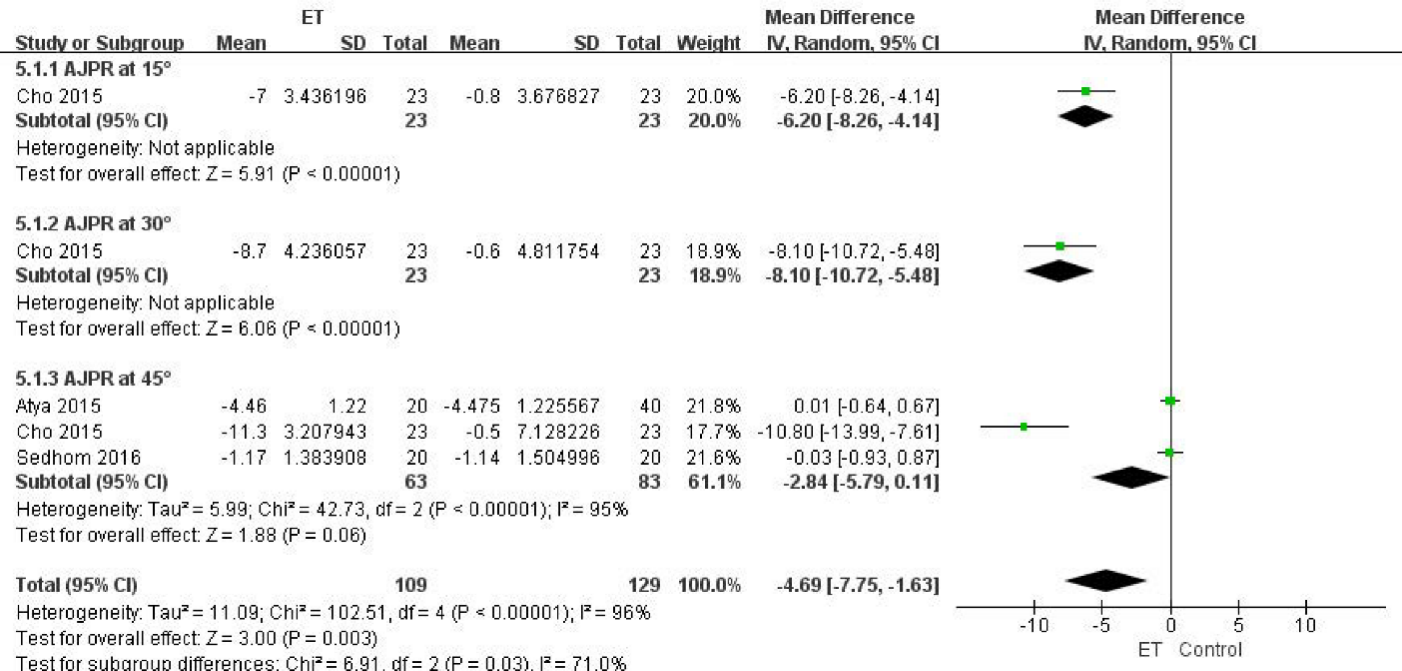
Proprioceptive Sensibility for ET compared with other forms of treatment.

Proprioceptive sensibility was assessed by testing the joint position sense through the Biodex system dynamometer (Biodex Corporation) in three trials [25, 31, 32]. Because three different target angles were set here we categorized this outcome according to the target angles set as 15 [25], 30 [25], and 45 [25, 31, 32] degrees respectively. No difference was found at target angles of 45 degrees (MD -2.84, 95% CI, -5.79 to 0.11; *P*=0.06), while for other two target angles, differences between the two groups were significant (For 15 degrees: MD -6.2, 95% CI, -8.26 to -4.14; *P*<0.001. For 45 degrees: MD -8.1, 95% CI, -10.72 to -5.48; *P*<0.001) (Fig. 7). The overall difference was also reported according to the proprioceptive sensibility (MD -4.69, 95% CI, -7.75 to -1.63; *P*=0.003; *P* for heterogeneity<0.001, *I^2^*=96%).

### Subgroup analysis

In our meta-analysis, significant heterogeneities for all outcomes were observed when ET was compared with other types of treatments. For the sub-group analysis, the self-reported pain during activity (*I^2^*=75.5%), knee ROM (*I^2^*=86.3%) and proprioceptive sensibility (*I^2^*=71%) data showed significant heterogeneity between the treatment effects of three subgroups. Others showed no significant heterogeneity between the treatment effects of the different durations of intervention (*I^2^* ranged from 0% to 34%). Both high risk and unclear risk trials were the cause of the heterogeneity in the self-related pain during activity category [24, 27, 29, 30-32], as well as in the proprioceptive sensibility [25, 31]. Otherwise, two outlying trials were the cause of the heterogeneity in the knee ROM category [24,31]. When these trials were excluded from the analysis, the results remained significant for both outcomes, but the tests for between subgroup heterogeneity were no longer significant (for pain during activity: MD -0.88, 95% CI, -1.62 to -0.13; P =0.02, *I*^2^=0%; for knee ROM: MD 6.68, 95% CI, 2.79 to 10.57; P =0.0008, *I^2^*=0%).

## DISCUSSION

This systematic review synthesized the evidence for ET effectiveness in a population with knee OA. ET is proved to be superior to other forms of intervention for the reduction of pain during activity, knee flexibility enhancement, knee-related health status improvement, and proprioceptive sensibility amelioration, but not to be more effective in increasing knee muscle strength related to knee OA.

Previous reviews have assessed the effects of ET on pain and disability in participants suffering from neurological and lymphatic pathologies [13], and also in individuals with other musculoskeletal disorders and sports injuries [12, 15, 35, 36], but none have examined its effects on patients with knee OA. We used a highly systematic search strategy to identify trials in all major databases following the recommendations from the Cochrane Collaboration [37]; therefore, the searches comprehensively identified most of the current evidence focused on ET for knee OA. Statistical significance was reported in four from five reported outcomes: self-related pain (during activity), knee flexibility, knee-related health status (WOMAC scale), and proprioceptive sensibility. Most of the significant differences we observed were at the short-term follow-up period. Only the improvement of knee-related health status assessed by the WOMAC scale was seen at long-term follow-up.

Although our study failed to find the effectiveness of ET on muscle strength, Anandkumar et al. conducted isokinetic testing 30 minutes after the ET application, and the results did show an increase in muscle strength for the ET group [24]. By contrast, when we focused on another study in which ET was compared with sham taping, no evidence of improvement in pain relief and WOMAC scale was shown [27]. All assessments reported by Anandkumar et al. were performed after the removal of the ET, to ensure that the assessor was blinded to group allocation, while in the other study it was unclear whether the assessments were performed with the ET on the skin, or after the removal of the tape. As it is unclear from the ET manufacturers whether the possible benefits of ET should only be expected while the tape is on [10], the effect of ET is likely to be influenced by whether it was on the skin or not. Besides, we speculated the various severity of knee OA may also contribute to the inconsistent finding, although we failed to find available evidence about it.

In this meta-analysis, we reviewed all the evidence from large numbers of trials, and evaluated the overall effect of ET on knee OA for different intervention durations. Totally, even though the brands of elastic therapeutic tape were various, we found that all studies reported elastic therapeutic taping for knee osteoarthritis have made use of the kinesio tape rather than other brands. What’s more, the methodological quality of the included trials in this meta-analysis was varied. Despite the several benefits of registering a clinical trial [38], only one included trial was registered [33]. Six of the 11 trials provided detailed information on sample size calculation [24-27, 29, 30]. The quality of evidence (GRADE) for all outcome measurements was inconsistent and ranged from moderate to very low quality (see Appendix S2), which means that in the future, data which are robust and have low risk of bias are likely to overturn some of the results of the interventions assessed in this meta-analysis.

As the conclusions from this meta-analysis are based on a number of studies which were not very rigorous, we cannot resolve all the issues involved in understanding the effects of ET in knee OA in one article. However, large-scale studies with long-term follow-up should be performed to confirm the effectiveness of ET for knee OA. First, a disciplined approach to randomization should be adopted, and the intervention allocation should be adequately concealed. Second, further studies should have well-designed methodologies, particularly with regard to the details of ET methods: none of the included studies lasted for more than 6 months. Third, studies should investigate the possibility that the length of intervention duration may affect the relative performance of ET compared to other interventions in managing knee OA. Fourth, outcome measures for further trials might usefully focus on pain intensity, functional performance, self-reported status, and adverse events. Furthermore, long-term follow-up would be useful to estimate whether any improvements owing to ET are persistent, and if so, for how long.

In conclusion, significant improvements were found in self-reported pain during activity, knee flexibility, knee-related health status, proprioceptive sensibility, and muscle strength compared with other forms of treatments. However, this analysis showed no difference between the experimental group and control group for knee muscle strength even though clinically significant difference was shown in the study of Dhanakotti et al [29]. Nevertheless, interpretation of our results has to be cautious because of the limitations of the included trials, such as methodological drawbacks and poor data quality.
